# Effects of total thoracoscopic surgery on coronary artery fistulae complicated with ectasia

**DOI:** 10.12669/pjms.321.7501

**Published:** 2016

**Authors:** Hongbin Sun, Liping Zhang, Xiuli Han, Zhongyu Wang, Lei Xu

**Affiliations:** 1 Hongbin Sun, Department of Thoracic Surgery, China-Japan Union Hospital of Jilin University, Changchun 130031, Jilin Province, PR China; 2 Liping Zhang, Department of Thoracic Surgery, China-Japan Union Hospital of Jilin University, Changchun 130031, Jilin Province, PR China; 3 Xiuli Han, Qianwei Hospital of Jilin Province, Changchun 130012, Jilin Province, PR China; 4 Zhongyu Wang, Department of Cardiology, China-Japan Union Hospital of Jilin University, Changchun 130031, Jilin Province, PR China; 5 Lei Xu, Department of Thoracic Surgery, 208th Hospital of People’s Liberation Army Chinese, Changchun 130000, Jilin Province, PR China

**Keywords:** Thoracoscopy, Coronary artery fistula, Coronary artery ectasia, Complication, Stress reaction

## Abstract

**Objective:**

To compare the effects of thoracotomy and total thoracoscopic surgery on coronary artery fistulae complicated with ectasia.

**Methods:**

Forty-six patients with coronary artery fistulae complicated with ectasia were randomly divided into a treatment group and a control group (n=23) which were given totally thoracoscopic surgery and thoracotomy respectively. Both groups were followed up.

**Results:**

All patients survived the surgeries and were discharged from the hospital. The treatment group had significantly less intraoperative blood loss, earlier postoperative ambulation and shorter postoperative hospitalization stay more than those of the control group (P<0.05). The two groups had similar plasm cortisol and adrenocorticotropic hormone levels one day before and after surgery. These levels peaked on the postoperative 3rd day, but those of the treatment group were significantly lower (P<0.05). The two groups had similar left and right atrial diameters as well as left ventricular ejection fractions (LVEFs) before surgery, but the treatment group had significantly higher postoperative LVEF (P<0.05) as well as significantly smaller left and right atrial diameters in the postoperative 1st and 3rd months (P<0.05). The treatment group was significantly less prone to postoperative complications such as chest pain, atelectasis and pulmonary infection than the control group (P<0.05).

**Conclusion:**

Total thoracoscopic surgery promoted the recovery of coronary artery fistulae complicated with ectasia, improved cardiac remodeling and cardiac function, and alleviated stress reaction, with well-proved safety.

## INTRODUCTION

Coronary artery fistula (CAF), as an abnormal connection between major coronary arterial branches or/and other branches, macrovessels and chambers, shows clinical symptoms in 80% of the patients over 20 years of age.^1^ An epidemiological study showed that the incidence of CAF complicated with coronary artery ectasia (CAE), as a rare congenital heart disease, was only about 0.30%.^2^ As a rare, congenital coronary artery anomaly, CAF is usually complicated with CAE, which lacks obvious early signs. Manifested as dyspnea and chest pain at the late stage, patients often claim myocardial ischemia before visiting hospitals.^3^

CAF results from dysplasia of intra myocardial trabecular sinusoids at the embryonic stage, which induces stenosis or occlusion and then connection between coronary arteries and chambers through epithelial tubes after birth.^4^ Besides, CAF starts from the left, right or bilateral coronary arteries and their branches (the right coronary artery in most cases), and has several endpoints in the injured chamber. During diagnosis, routine angiography may fail to display the distal end of the right coronary artery or whether it converges in the chamber owing to large aneurysms and blood circulation disorders. As a result, right ventricular fistulae can only be revealed during surgery.^5^

According to the diameters of involved blood vessels, there are large and small fistulae. Large fistulae evidently affect patients both physically and psychologically by leading to abnormal cardiac morphology and structures.^6^ Small fistulae have even poorer prognosis, because they are often found by coronary angiography after being suspected as coronary artery disease due to the lack of obvious symptoms.^7^

In clinical practice, CAF complicated with CAE is mainly treated by surgery and transcatheter interventional therapy. Although transcatheter occlusive therapy is a state-of-the-art method, it has not been widely applicable to surgery owing to troublesome disassembly of balloons, membrane-covered stents and coils, as well as high operational requirements.^8^-^10^ Currently, minimally invasive, video-assisted thoracoscopic surgeries have been extensively performed,^11^ especially totally thoracoscopic surgery. This method barely injures the chest wall muscles, minimizes postoperative pain, allows quick recovery, prevents hypersensitivity-induced damages and slightly affects organs and immune function.^12^,^13^

In this study, we compared the effects of thoracotomy and total thoracoscopic surgery on CAF complicated with CAE, aiming to verify the advantages of this strategy.

## METHODS

### Subjects

This study was approved by the ethics committee of our hospital, and written consent was obtained from all patients. Forty-six patients with CAF complicated with CAE, who were treated in our hospital from February 2008 to June 2014, were selected.

### Inclusion criteria

Large fistulae were diagnosed by routine transthoracic echocardiography (TTE), 12-lead synchronization electrocardiogram and chest X-ray; fistula diameter >5 mm; age ≥3 years old, body weight ≥10 kg; CAE was pathologically diagnosed; with written consent; suitable for surgery or interventional therapy; with normal liver, kidney functions, myocardial enzymatic activity and coagulation.

### Exclusion criteria

Extreme tortuosity of involved blood vessels; right heart catheterization disclosed left-right shunt and severe pulmonary hypertension; with surgical contraindications such as stroke and severe traumas before hospitalization.

The patients were randomly divided into a treatment group and a control group (n=23). The treatment group consisted of 13 males and 10 females who were aged from 6 to 28 years old (average: 17.23±2.44), with the average body mass index (BMI) of 22.22±2.93 kg/m^2^; complicated diseases: 6 cases of hypertension, 7 cases of diabetic mellitus and 3 cases of bronchitis. Disease types: 10 cases of right coronary artery-right ventricular fistulae, 12 cases of right coronary artery-left ventricular fistulae and 1 case of left coronary artery-right atrial fistula; clinical symptoms (individual or complicated): 14 cases of palpitations and shortness of breath, 15 cases of chest pain, 9 cases of dizziness and 2 cases of syncope.

The control group comprised 13 males and 10 females who were aged from 6 to 28 years (average: 17.22±2.13 years), with the average BMI of 22.13±3.13 kg/m^2^; complicated diseases: 8 cases of hypertension, 4 cases of diabetic mellitus and 4 cases of bronchitis. Disease types: 10 cases of right coronary artery-right ventricular fistulae, 10 cases of right coronary artery-left ventricular fistulae and 3 cases of left coronary artery-right atrial fistula; clinical symptoms (individual or complicated): 12 cases of palpitations and shortness of breath, 14 cases of chest pain, 8 cases of dizziness and 1 case of syncope. The two groups had similar gender ratio, age, BMI, complicated diseases, clinical symptoms and disease types (P>0.05).

### Treatment methods

### Control group

This group was subjected to routine thoracotomy, i.e. suturing of the fistula orifice, ligation of fistula branches as well as coronary arterial incision and repair. Their vital signs were monitored simultaneously.

### Treatment group

Under direct vision, total thoracoscopic surgery was performed, with vital signs monitored simultaneously. Patient in the standard lateral position was sterilized on the chest skin and subjected to inhalation anesthesia. Three to four incisions sized about 1.5 cm were made, and skin and subcutaneous tissues were cut open sequentially to insert a thoracoscope. Separation and incision were made with a suction pump, after which suturing of the fistula orifice, ligation of fistula branches as well as coronary arterial incision and repair were carried out. Afterwards, the wound surface was hemostasized thoroughly.

After the surgery, the patients were given antibiotics to prevent infections and orally administered with 100 mg/daily aspirin for 6 consecutive months to prevent thrombosis. Their clinical symptoms and vital signs were closely monitored, and symptomatic interventions were performed when necessary.

### Observation indices

### Perioperative indices

Surgical time, intraoperative blood loss, postoperative ambulation time and hospitalization stay length were recorded.

### TTE indices

The size of each atrium and cardiac functions were examined before and three days, one month and six months after surgery. TTE was completed by experienced personnel, and cardiac functions were detected based on left ventricular ejection fraction (LVEF).

### Complications

Postoperative complications, such as chest pain, atelectasis and pulmonary infection, were followed up in the postoperative 6th month.

### Stress reaction

Fasting venous blood (5 ml) was collected in the early morning of one day before and one day, three days after surgery, and centrifuged at 3000 rpm for 5 minutes, from which the plasma was collected and stored in a -40°C refrigerator. Cortisol (COR) and adrenocorticotropic hormone (ACTH) levels in the plasma were detected with a radioimmunoassay kit (Beijing Fluorine Bio-tech Co., Ltd.).

### Statistical analysis

All data were analyzed by SPSS19.0. The categorical data were compared by using repeated measures analysis of variance, Fisher’s least significant difference method and t-test for two independent samples. The numerical data were compared with χ^2^ analysis. P<0.05 was considered statistically significant.

## RESULTS

### Comparison of perioperative indices

All patients survived the surgeries and were discharged from the hospital. The treatment group had significantly less intraoperative blood loss, earlier postoperative ambulation and shorter postoperative hospitalization stay length than those of the control group (P<0.05) (Table-I).

**Table-I:** Perioperative indices (x±s).

Group	Case No. (n)	Surgical time (min)	Intraoperative blood loss (ml)	Postoperative ambulation (d)	Postoperative hospitalization stay length (d)
Treatment group	23	149.36±14.13	60.39±6.33	4.13±0.44	10.63±0.34
Control group	23	147.35±15.34	120.31±8.74	7.33±0.21	17.83±0.72
t		0.123	8.453	6.442	9.987
P		<0.05	<0.05	<0.05	<0.05

### Comparison of TTE indices

The two groups had similar left and right atrial diameters as well as LVEFs before surgery, but the treatment group had significantly higher postoperative LVEF (P<0.05) and significantly smaller left and right atrial diameters in the postoperative 1st and 3rd months (P<0.05) (Table-II).

**Table-II:** TTE indices at different time points (x±s)

Time	Treatment group (n=23)			Control group (n=23)		

	Right atrial diameter (mm)	Left atrial diameter (mm)	LVEF (%)	Right atrial diameter (mm)	Left atrial diameter (mm)	LVEF (%)
Before	28.10±2.11	29.80±1.77	49.28±4.33	28.12±3.09	29.83±2.11	49.38±5.12
Three days after	28.71±2.30	29.29±3.11	57.78±5.33*#	28.45±2.73	29.55±4.23	51.93±5.33
One month after	27.28±1.99	26.01±2.12*#	63.92±5.44*#	27.83±3.12	28.77±3.09	57.33±6.43*
Three months after	28.24±2.10	26.00±1.83*#	64.87±4.22*#	27.55±2.78	28.56±2.78	57.89±4.33*

# Compared with the control group, P<0.05;

* compared with the data before surgery, P<0.05.

### Comparison of postoperative complications

The treatment group suffered from significantly less postoperative complications such as chest pain, atelectasis and pulmonary infection than the control group did (P<0.05) (Table-III). All complications were mitigated by symptomatic interventions.

**Table-III:** Incidence of postoperative complications (case, %).

Group	Case number (n)	Chest pain	Atelectasis	Pulmonary infection	Total
Treatment group	23		1 (4.3%)	0 (0.0%)	1 (4.3%)	2 (8.6%)
Control group	23		3 (13.0%)	1 (4.3%)	3 (13.0%)	7 (30.4%)
χ^2^			3.112	0.392	3.112	18.241
P			<0.05	>0.05	<0.05	<0.05

### Comparison of stress reaction indices

The two groups had similar plasma COR and ACTH levels one day before and after surgery. These levels peaked on the postoperative 3rd day, but those of the treatment group were significantly lower (P<0.05) (Table-IV).

**Table-IV:** Plasma stress reaction indices (ng/ml, x±s)

Group	Case No. (n)	COR			ACTH		

		One day before	One day after	Three days after	One day before	One day after	Three days after
Treatment group	23	68.34±10.21	93.21±16.34*#	113.00±18.32*#	3.21±0.39	3.33±0.64#	3.36±0.18#
Control group	23	69.15±13.43	94.34±15.32*	155.33±14.93*	3.23±0.31	3.91±0.73*	4.15±0.94*

# Compared with the control group, P<0.05;

* compared with the data before surgery, P<0.05.

## DISCUSSION

CAF complicated with CAE is pathophysiologically typified by increase in the blood volume of the coronary circulation and left-right shunt or coronary bypass to the left ventricle, which can induce chamber enlargement and heart failure in addition to obvious heart murmurs.^14^ Similarly, all enrolled patients in this study suffered from palpitations, breath shortness and dizziness, but they rarely fainted.

Traditionally, patients are treated by thoracotomy, i.e. suturing of the fistula orifice, ligation of fistula branches as well as coronary arterial incision and repair. However, this protocol has many disadvantages such as major trauma, high surgical risk and slow postoperative recovery. Regardless, patients are seldom misdiagnosed by using this method that is also suitable for treating those with involved blood vessels diametered ≥10 mm.^15^

In contrast, total thoracoscopic surgery, which is suitable for the patients having involved blood vessels diametered 5-10 cm, can replace thoracotomy because of small surgical incision, short hospitalization stay and rapid postoperative recovery.^16^ Particular attention should be paid to intraoperative exploration and postoperative intrathoracic gas evacuation. Damage or ligation of related arteries should be prevented if possible. All patients survived the surgeries and discharged from the hospital, well-recovered without any deaths. The treatment group had significantly less intraoperative blood loss, earlier postoperative ambulation and shorter postoperative hospitalization stay than those of the control group (P<0.05).

In general, suturing of the fistula orifice and ligation of fistula branches are mainly performed to disconnect the proximal fistula end, while coronary arterial incision and repair are conducted for the distal end.^17^ Fistulae, including multiple ones, should be closed by using mattress sutures. After surgery, patients should be given anti-infective, antiplatelet and anticoagulant therapies to prevent possible complications.^18^ The two groups were followed up for 6 months, and the treatment group was significantly less prone to postoperative complications such as chest pain, atelectasis and pulmonary infection than the control group (P<0.05). Meanwhile, complications of the treatment group were alleviated by symptomatic treatment, suggesting that totally thoracoscopic surgery caused fewer damages to patients.

CAF complicated with CAE accounts for 8% of congenital coronary artery anomalies, which may not be diagnosed with TTE and thus needs further coronary angiography.^19^ TTE can directly displays the origin of dilated coronary artery and trace it to reveal the location and number of fistulae and to estimate the shunt volume, providing considerable diagnostic value for CAF. Meanwhile, TTE is the most accessed follow-up method for determining prognosis.^20^ The two groups had similar left and right atrial diameters as well as LVEFs before surgery, whereas the treatment group had significantly higher postoperative LVEF (P<0.05) and significantly smaller left and right atrial diameters in the postoperative 1st and 3rd months (P<0.05). Hence, although the low shunt volume of fistula did not evidently injure cardiac functions in a short time, the chambers and ventricles of the treatment group shrank at all selected postoperative time points compared with those before surgery. Given the increased LVEF simultaneously, this minimally invasive surgery benefited cardiac remodeling.

Under stress, the hypothalamic-pituitary-adrenal axis and the sympathetic-adrenal- medullary axis are activated. The former increases the secretion of ACTH and COR^13^, and the latter raises heart rate and cardiac output as well as redistributes blood circulation. Accordingly, increase in the COR level indicates the onset of stress reaction and trauma.^21^ In this study, the two groups had similar plasma COR and ACTH levels one day before and after surgery, which reached maxima on the postoperative 3rd day, but the levels of the treatment group were significantly lower (P<0.05). The results indicated total thoracoscopic surgery was conducive to recovery due to smaller traumas and milder stress reaction.

In summary, total thoracoscopic surgery facilitated the recovery of CAF complicated with CAE, improved cardiac remodeling and cardiac function, and safely mitigated stress reaction.
